# Direct calculation of variable-resolution maps by fully analytic approximations to atomic images

**DOI:** 10.1107/S1600576725010167

**Published:** 2025-11-26

**Authors:** Alexandre G. Urzhumtsev, Vladimir Y. Lunin

**Affiliations:** a Centre for Integrative Biology Department of Integrated Structural Biology IGBMC (Institute of Genetics and of Mol­ecular and Cellular Biology) Centre National de la Recherche Scientifique (CNRS) UMR 7104/Institut National de la Santé de la Recherche Médicale (Inserm) U964/Université de Strasbourg 1 rue Laurent Fries 67404Illkirch France; b Université de Lorraine Physical Department Vandoeuvre-lès-Nancy 54506 France; c Institute of Mathematical Problems of Biology RAS Keldysh Institute of Applied Mathematics of Russian Academy of Sciences Pushchino 142290 Russian Federation; DESY, Hamburg, Germany

**Keywords:** cryo-EM, variable-resolution maps, direct map calculation, local resolution, shell functions, analytic approximation, computer programs

## Abstract

Crystallographic maps are typically computed as Fourier series using complex-valued structure factors, with a single set of coefficients applied across the entire region and associating a single resolution value with the whole map. In cryo-electron microscopy, however, experimental maps often have variable local resolution. As a consequence, applying the same procedure to compute model maps for comparison with the respective experimental ones becomes complicated. An alternative strategy involves representing model maps as sums of atomic images. They are oscillatory functions of the distance from the atomic center and depend on atomic displacement parameters and local resolution. To calculate such maps, we propose a stand-alone program which uses the shell-function approximation of atomic images, making them analytic functions of all their parameters including the local resolution associated with each atom.

## Abstract 2

Crystallographic and cryo-electron microscopy maps with variable resolution can be calculated as a sum of analytic shell-function approximations to atomic images. To implement this procedure, a stand-alone Python script, *ModQMap*, has been developed.

## 1. Introduction

Crystallographic maps are typically computed as Fourier series using complex-valued structure factors, with a single set of coefficients applied across the entire map. Consequently, a uniform resolution is usually assigned to the map, although directional anisotropy in resolution can be analyzed (*e.g*. Urzhumtseva *et al.*, 2013 ▸; Tickle *et al.*, 2016 ▸; and references therein). An alternative approach involves computing the map as a sum of atomic contributions at limited resolution. While this procedure complicates the inclusion of non-atomic components such as bulk solvent, it offers several advantages. Notably, it is particularly well suited for generating maps of electrostatic scattering potential in cryo-electron microscopy (cryo-EM), where resolution often varies across different regions (*e.g*. Cardone *et al.*, 2013 ▸), leading to the concept of local resolution (Kucukelbir *et al.*, 2014 ▸; Vilas *et al.*, 2018 ▸; and others).

These atomic contributions are atomic images computed at a given resolution *d*, with corresponding atomic displacement parameter (ADP) and occupancy values. Therefore, to reproduce the experimental map as a sum of such images, each atom of the model must be assigned an additional parameter, namely the local resolution of the map in its vicinity (Urzhumtsev & Lunin, 2022*a* ▸).

The atomic images used to calculate a map are oscillating functions of the distance from the atomic center. These images, or rather approximations to them, can be computed either numerically or as analytic functions of ADP at a given resolution (*e.g*. Diamond, 1971 ▸; Lunin & Urzhumtsev, 1984 ▸; Chapman, 1995 ▸; Mooij *et al.*, 2006 ▸; Chapman *et al.*, 2013 ▸; DiMaio *et al.*, 2015 ▸; Sorzano *et al.*, 2015 ▸; Pintilie *et al.*, 2020 ▸; and references therein). When the atomic scattering factors are approximated by a sum of Gaussian functions (*e.g*. Doyle & Turner, 1968 ▸; Agarwal, 1978 ▸; Waasmaier & Kirfel, 1995 ▸; Peng, 1999 ▸; Grosse-Kunstleve *et al.*, 2004 ▸; Brown *et al.*, 2006 ▸), the corresponding atomic images can be written as analytic functions of both ADP and resolution (Urzhumtsev & Lunin, 2022*a* ▸). This can be done by representing them as series of especially developed spherically symmetric three-dimensional functions, 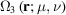, which depend on two scalar parameters, 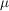 and 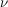 (Urzhumtsev & Lunin, 2024 ▸), defining the radius and the width of the spherical shell within which the function values are concentrated.

This manuscript presents a stand-alone program that calculates maps with potentially variable resolution across the regions as a sum of analytic approximations to atomic images.

## 2. Methods and algorithms

### 2.1. Shell functions

The approximation of atomic scattering factors, and thus atomic densities, by Gaussian functions is important because it renders them analytic functions of ADP. This property arises from the invariance of the class of Gaussian function under convolution with other Gaussians. However, atomic images at limited resolution, which are inherently oscillatory in three-dimensional space, cannot be approximated by Gaussian functions. Instead, they can be represented by shell functions 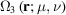, a class of spherically symmetric functions whose values are concentrated within a shell of prescribed width 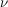 centered at distance 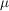 from the origin. This class is also invariant under convolution with Gaussians, preserving analytic dependence on ADP (Urzhumtsev & Lunin, 2024 ▸). Gaussian functions represent a particular case of 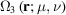 with 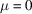.

By approximating the interference function in three-dimensional space with shell functions and the atomic scattering factors with Gaussians, atomic images can be expressed as analytic functions of all atomic parameters, including the local resolution *d* associated with each atom (Urzhumtsev & Lunin, 2022*a* ▸). While this approach is computationally more demanding than directly using shell approximations to atomic images precomputed at a fixed resolution, it enables a more detailed analysis of the model-to-map correspondence. It also opens the possibility for analytic refinement of local resolution values, now associated with atoms. Although potentially less practical for fast refinement procedures with fixed resolution, this method offers substantial benefits for advanced model assessment and resolution mapping.

### 2.2. Procedure and its parameters

To implement the procedure described above, a stand-alone program, *ModQMap*, has been developed. The program takes as input an atomic model and a file containing the respective atomic scattering factors approximated by Gaussian functions. On the basis of this information, it generates a map of the corresponding distribution, such as electron density or electrostatic scattering potential.

Each atom, in addition to its coordinates in the crystal (for crystallography) or pseudo-crystal (for cryo-EM), occupancy, and ADP, is assigned an individual value of local resolution, *d*. This local resolution can be stored directly in the atomic records. Alternatively, it may be assigned as a global value shared by all atoms, which is convenient for crystallographic applications, or extracted from a map of local resolution (*e.g*. Dai *et al.*, 2023 ▸). In the latter case, the local resolution at the nearest grid point is assigned to each atom.

The procedure employs the 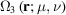 approximation to the three-dimensional normalized interference function 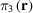. as described by Urzhumtsev & Lunin (2024 ▸). Using this approximation, each atomic image is computed within a spherical region of radius *R*_max_ centered at the atomic position. Increasing *R*_max_ improves the map accuracy but increases the computation time. Previous analyses (Urzhumtsev *et al.*, 2022 ▸) have shown that a better accuracy can be achieved by choosing *R*_max_ as an integer or half-integer multiple of the resolution *d* associated with the given atom, *R*_max_ = *kd*. For accurate maps, a factor *k* equal to 2.0 or 2.5 is typically sufficient (Urzhumtsev & Lunin, 2022*b* ▸). Since each 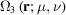 term contributes within a spherical shell of finite width, a few additional terms with μ > *R*_max_ should be included to ensure map accuracy.

The grid parameters used for map calculation, namely, the map boundaries and grid step, can be either specified directly in the input parameters or taken from a reference (experimental) map, if provided.

Although cryo-EM calculations are typically carried out within orthogonal pseudo-cells, the procedure is general and supports maps and models defined in arbitrary unit cells. However, the current version of the program does not apply symmetry operations. Therefore, all atoms contributing to the computed portion of the map must be explicitly included in the input atomic model.

The calculated map can be computed in absolute or in sigma-valued scales, or be optimally scaled to match the experimental map either globally or within the molecular region defined as the set of grid points where the model contributes.

If an experimental map is provided, the program automatically compares the calculated and experimental maps, both over the entire volume and within the molecular region.

Additionally, if a map-like file of local resolution is supplied, the program can generate an output model file in which each atom is annotated with its corresponding local resolution value. This facilitates downstream analysis and further computations.

## 3. Software

### 3.1. System and software requirements

The program is stand-alone and does not require installation. It is written in basic Python3 and relies on the standard *NumPy* library (https://numpy.org/). The program, along with associated data files and an example, is freely available upon request from one of the authors (AGU) and from the site https://git.cbi.igbmc.fr/sacha/mqm-calculate-variable-resolution-maps.

### 3.2. Input data

The program requires a text file, ModQMap.dat, containing input parameters that specify both the input/output files and the calculation settings. All parameters are defined by keywords, and default values are available for most of them. An example input file is provided, including the full list of parameters and explanatory comments on their usage, and possible and default values.

The atomic model file can be provided in either Protein Data Back (PDB; Berman *et al.*, 2020 ▸; https://www.rcsb.org/) or CIF format. Atomic records can be completed by the values of the associated local resolution. If these values are available for individual atoms, they can be included directly in the model file. For CIF format, currently they should be specified using the keyword _atom_site.resolution; for PDB format, the resolution values should occupy columns 67–72 of the atomic records (otherwise free except for special cases). Unit-cell parameters may be defined or redefined via ModQMap.dat.

Two more input files with standard information are required. These files are maintained externally from the program, making it straightforward to update or replace them as needed without modifying the code. The first file contains coefficients of Gaussian approximations to the atomic scattering factors. Several versions of this file are provided, and the desired one can be selected via input parameters. The computation time is approximately proportional to the number of Gaussians used in the approximation. However, while simpler approximations may reduce computation time, they may compromise map accuracy.

The second file contains the coefficients for the 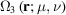 approximation of the three-dimensional interference function as given by Urzhumtsev & Lunin (2024 ▸). The current approximation to the interference function enables calculation of atomic images up to a distance of *R*_max_ = 20*d*, where *d* is the local resolution.

Optionally, the user can provide an experimental map and a local resolution map as additional inputs, both either in MRC (https://www.ccpem.ac.uk/mrc-format/) or in *CNS* for­mat (Brünger *et al.*, 1998 ▸; Brunger, 2007 ▸), enabling further comparison and analysis.

### 3.3. Output data

The calculated map ρ^calc^ can be saved either in MRC or in *CNS* format.

The program creates a log file which mirrors input information and provides various statistics of the calculated map. If a control map ρ^obs^ is available, the program communicates also the values of the maps’ correlation and of their discrepancy
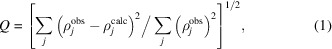
with the sums over grid nodes calculated both for the whole map and inside the model mask. These values are computed for the calculated map taken in different scales.

If requested by the input parameters, the atomic model completed with the associated local resolution values can be output in the same format as the input model, either PDB or CIF.

## 4. Practical application to macromolecular studies

**Figure 1:**
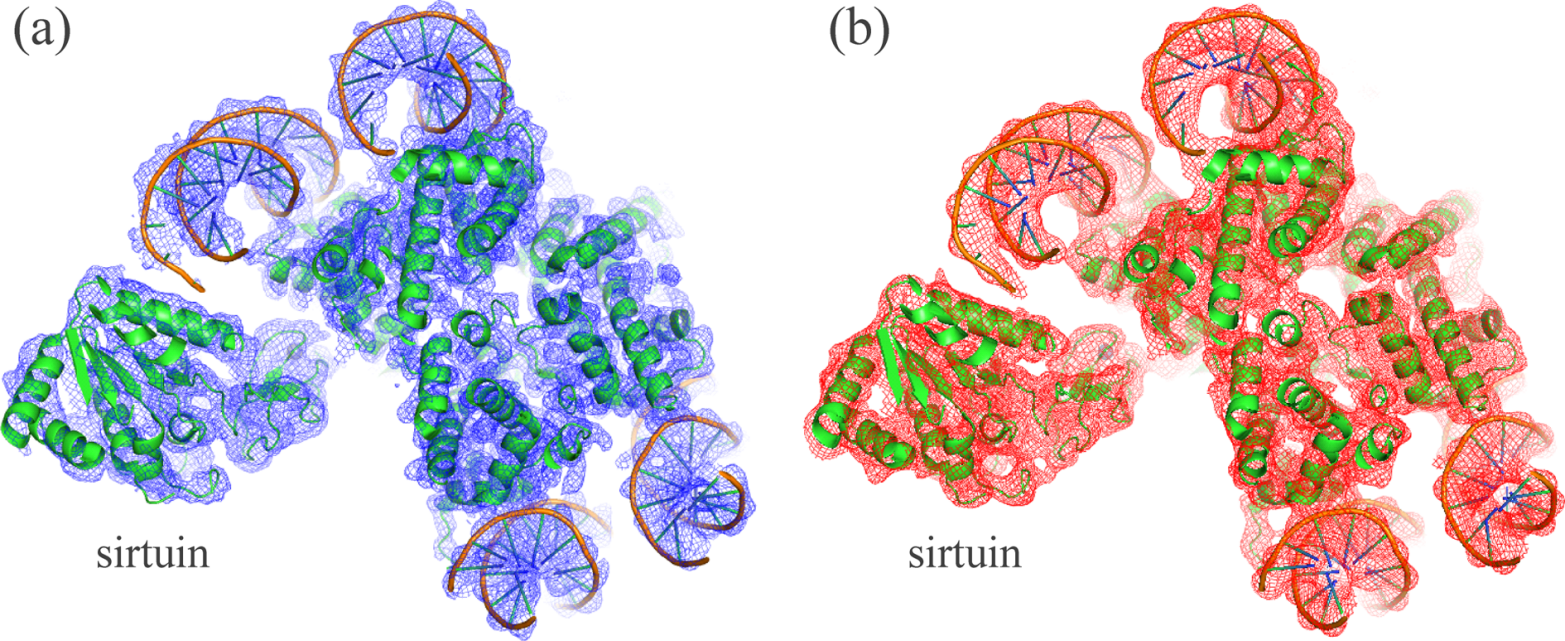
Experimental (*a*) and calculated (*b*) cryo-EM variable-resolution maps of the nucleosome–sirtuin complex. Figure was prepared with *PyMOL* (Schrödinger & DeLano, 2020 ▸).

**Figure 2:**
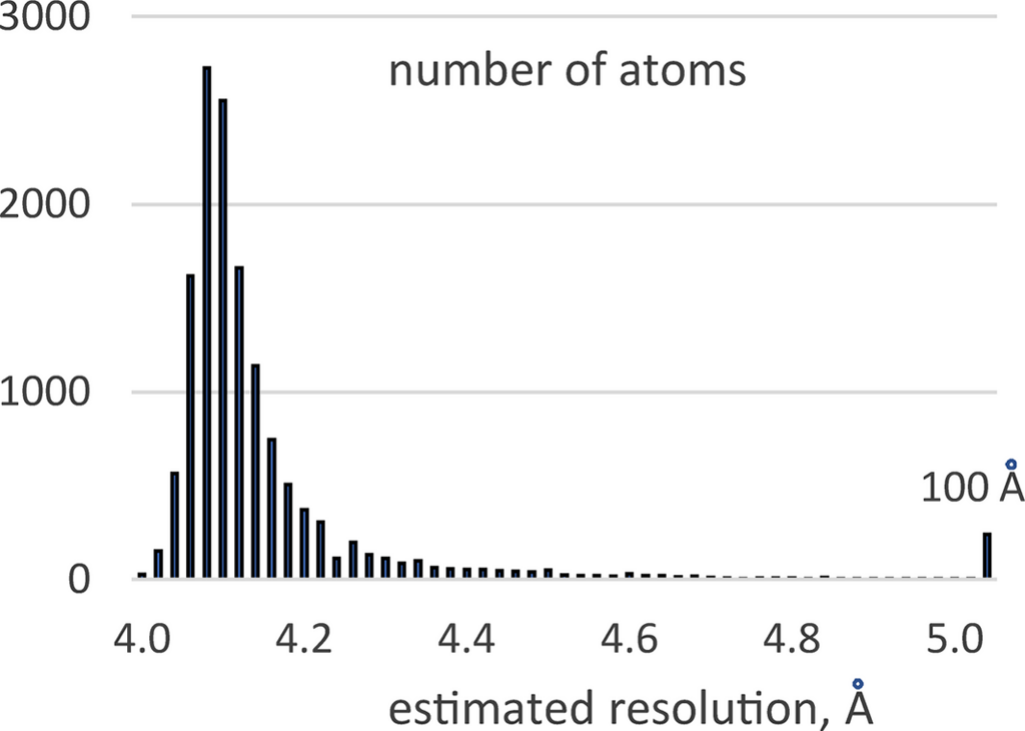
Distribution of the local resolution values associated with the model atoms of the nucleosome–sirtuin complex.

The structure of the nucleosome–sirtuin complex (PDB entry 8of4) comprises nine protein chains and two nucleic acids, totaling over 14000 non-hydrogen atoms (Smirnova *et al.*, 2023 ▸). The corresponding composite map of the electrostatic scattering potential [entry EMD-16845; Electron Microscopy Data Bank (EMDB; The wwPDB Consortium, 2024 ▸; https://www.ebi.ac.uk/emdb/)] exhibits pronounced resolution heterogeneity [Fig. 1 ▸(*a*)]. Local resolution estimates were obtained using *CryoRes* (Dai *et al.*, 2023 ▸), which provides such values in the form of a map on a regular grid identical to that for the 3D reconstruction. The resolution assigned to each atom was taken as the value of the local resolution map at the grid node nearest to the atomic center. As Fig. 2 ▸ shows, the local resolution values associated with atoms vary nearly continuously between 4 and 5 Å for the majority of atoms. For approximately 300 atoms, mainly located in the sirtuin region, *CryoRes* assigned a nominal local resolution of 100 Å, reflecting areas too blurred for reliable estimation.

These local resolution estimates differ from the nominal resolution of 2.94 Å reported at the EMDB site. This discrepancy can be attributed to the coexistence of different resolution definitions in cryo-EM. Some approaches are based on the similarity of Fourier coefficients calculated from two half-maps (FSC analysis), while others estimate resolution from the size of discernible map details (Appendix *A*[App appa]). The EMDB-reported value was derived from an FSC-based analysis, whereas the estimates reported by Dai *et al.* (2023 ▸) follow the latter approach; they are consistent with the values obtained by ourselves (Lunin *et al.*, 2023 ▸).

*ModQMap* is well suited to reproducing the experimental map for situations in which atomic images vary significantly depending on their displacement parameters and local resolution values. As an initial test, we performed a rapid, lower-accuracy calculation using *R*_max_ = *d* and replacing the *d* = 100 Å value (arbitrarily assigned by *CryoRes* to highly blurred regions) with 7 Å, lower than the resolution for relatively well structured regions and already sufficient to strongly blur the atomic images beyond these regions. This calculation required only about one minute at an ordinary PC. Fig. 1 ▸(*b*) shows the resulting map. Subsequently, we performed a higher-accuracy computation by increasing *R*_max_ to 2.5*d*. We also extended the upper resolution limit from 7 to 10 Å to ensure that no artificial map features were introduced by the corresponding atomic images. Both these changes had little impact on the overall statistical indicators of the map (not shown), suggesting that, in practice, including multiple spherical-shell terms is required for only high-precision calculations. Nonetheless, modeling at least a few of the Fourier ripples in the atomic images remains important both to assure map accuracy (Urzhumtsev & Lunin, 2022*a* ▸) and to recover the atomic displacement factor and refine associated local resolution values (Lunin & Lunina, 2025 ▸).

## 5. Discussion

Structural macromolecular studies require various computer tools adapted to different goals and answering different questions. While modern workflows rely on large software packages, stand-alone programs retain important advantages, most notably, offering users greater transparency into the underlying calculations and flexibility to tailor the software to specific needs. Naturally, such stand-alone tools are expected to remain compatible in file formats with major packages.

We present here an example of such a tool and one of its applications to macromolecular structure analysis, namely, cryo-EM studies of the nucleosome–sirtuin complex. Developed in Python rather than compiled languages like Fortran or C++, the program is not optimized for speed and is therefore not intended for integration into iterative refinement procedures. Instead, as the example shows, with its extensive parameterization, it is particularly well suited for methodological investigations and targeted analyses of experimental images in macromolecular crystallography and in cryo-electron microscopy.

## Appendix A Resolution of cryo-EM maps

In crystallography, the formal map resolution is primarily defined by the cut-off radius of the reflection set used for its calculation; this may lead to ambiguities when the dataset is incomplete (*e.g*. Urzhumtseva *et al.*, 2013 ▸). In contrast, data incompleteness is not an issue in cryo-EM, where the map resolution is assigned according to different criteria.

The first group of resolution measures is based on the comparative analysis of Fourier coefficients derived from two independent 3D reconstructions built from complementary subsets of 2D projections (van Heel *et al.*, 1982 ▸; Saxton & Baumeister, 1982 ▸; Harauz & van Heel, 1986 ▸; Van Heel, 1987 ▸) [more recently, Scheres (2012 ▸) and other work]. Several Fourier shell correlation (FSC) threshold choices have been proposed (Böttcher *et al.*, 1997 ▸; Beckmann *et al.*, 1997 ▸; Rosenthal & Henderson, 2003 ▸; van Heel & Schatz, 2005 ▸).

The second group of resolution estimates relies on the analysis of map features (*e.g*. Kucukelbir *et al.*, 2014 ▸; Dai *et al.*, 2023 ▸), making these estimates conceptually similar to those used in optics and crystallography. The internal confidence of Fourier coefficients, defined by FSC analysis as above, does not directly determine the size of map details addressed by the second group of the methods, although the two are correlated to some extent. A discussion of the assignment of nominal map resolution values (*e.g*. Penczek, 2010 ▸; van Heel & Schatz, 2017 ▸; Sorzano *et al.*, 2017 ▸; Afonine *et al.*, 2018 ▸) lies beyond the scope of this article.
